# Rider factors associated with severe injury after a light motorcycle crash: A multicentre study in an emerging economy setting

**DOI:** 10.1371/journal.pone.0219132

**Published:** 2019-06-28

**Authors:** Carlos Lam, Chih-Wei Pai, Chia-Chang Chuang, Yu-Chun Yen, Chia-Chieh Wu, Shih-Hsiang Yu, Kuo-Sheng Hung, Wen-Ta Chiu

**Affiliations:** 1 Emergency Department, Department of Emergency and Critical Care Medicine, Wan Fang Hospital, Taipei Medical University, Taipei, Taiwan; 2 Graduate Institute of Injury Prevention and Control, College of Public Health, Taipei Medical University, Taipei, Taiwan; 3 Department of Emergency Medicine, School of Medicine, College of Medicine, Taipei Medical University, Taipei, Taiwan; 4 Department of Emergency Medicine, National Chen Kung University Hospital, College of Medicine, National Chen Kung University, Tainan, Taiwan; 5 Research Center of Biostatistics, College of Management, Taipei Medical University, Taipei, Taiwan; 6 Institute of Transportation, Ministry of Transportation and Communications, Executive Yuan, Taipei, Taiwan; 7 Department of Neurosurgery, Wan Fang Hospital, Taipei Medical University, Taipei, Taiwan; University of British Columbia, CANADA

## Abstract

**Objective:**

In Taiwan, light motorcycles (LMCs) with cylinder capacities between 50 and 250 cc are widely used for daily commute. These vehicles are operated in a mixed traffic environment and prohibited on highways. In light of increasing motorcycle casualties, we conducted a multicentre study to analyse rider factors affecting injury severity.

**Methods:**

Riders hospitalised upon LMC crashes were contacted. Information on demographics, comorbidities, and riding behaviours was collected through questionnaires and linked to hospital data. The injury severity score (ISS) and length of hospitalisation (LOH) were used as injury severity measures.

**Results:**

In total, 725 patients (mean age: 37.7 years; 64% men) completed their questionnaires. Multivariate analysis results showed that age ≥ 65 years, half-face helmets, protective clothing, collisions with a bus/truck or car, and fatigue riding were risk factors for having an ISS of ≥9. Age ≥ 65 years; motorcycle crashes ≥2 times in the previous year; anaemia; rural crashes; half-face helmets; protective boots; collisions with a bus/truck, car, or a stationary object; alcohol/stimulating refreshment consumption; and fatigue riding were risk factors for increased LOH. A protective factor was individuals working in commerce. Collisions with opening car doors caused low risks of having an ISS of ≥9 and a short LOH.

**Conclusion:**

Certain factors were significantly associated with riders’ injury severity and related medical resource consumption. Because of differences in the power output, use, and riding environment, risk factors for severe injuries in LMC crashes are dissimilar from those for heavy motorcycles (cylinder capacities > 250 cc) in developed countries and deserve more attention for injury prevention. Further in-depth evaluation of significant factors based on this study’s results can yield valuable information to reduce severe injuries after LMC crashes in countries and areas with a high dependency on motorcycles, even considering the popularity of electric motorcycles.

## Introduction

Motorcycles are one of the most injury-causing road transportation vehicles [1]. Recently, the number of motorcycles has considerably increased in numerous countries, as reflected by the fact that the global sales volume of motorcycles in 2018 has increased by 1.8% compared with that in the previous year [2]. In addition to a motorcycle’s low price, usage convenience, and simple operating mechanisms, other factors, including increased urban traffic congestion, insufficient public transportation systems, and certain socioeconomic conditions, must also be considered.

The growth of motorcycles is fast in developing countries, such as Vietnam, India, Pakistan, and the Philippines [2], and the majority of motorcycles used there are light motorcycles (LMCs) with cylinder capacities between 50 and 250 cc. In general, LMCs are mainly used for transportation and not for recreational purposes. These vehicles are operated in a mixed traffic environment and are prohibited on highways. In countries where transportation is highly motorcycle dependent, motorcycle injuries have become a serious issue of road safety.

Many studies on injury severity after motorcycle crashes have been conducted in developed countries. However, motorcycles used in developed countries typically have cylinder capacities of >250 cc, are mostly for recreational use, and are allowed on highways. These motorcycles’ power output and use as well as their riding environment differ a lot from those in developing countries. Moreover, rider factors, such as demographics, health conditions, and riding behaviours, are considered to vary among countries at different phases of economic development.

**Fig 1:**
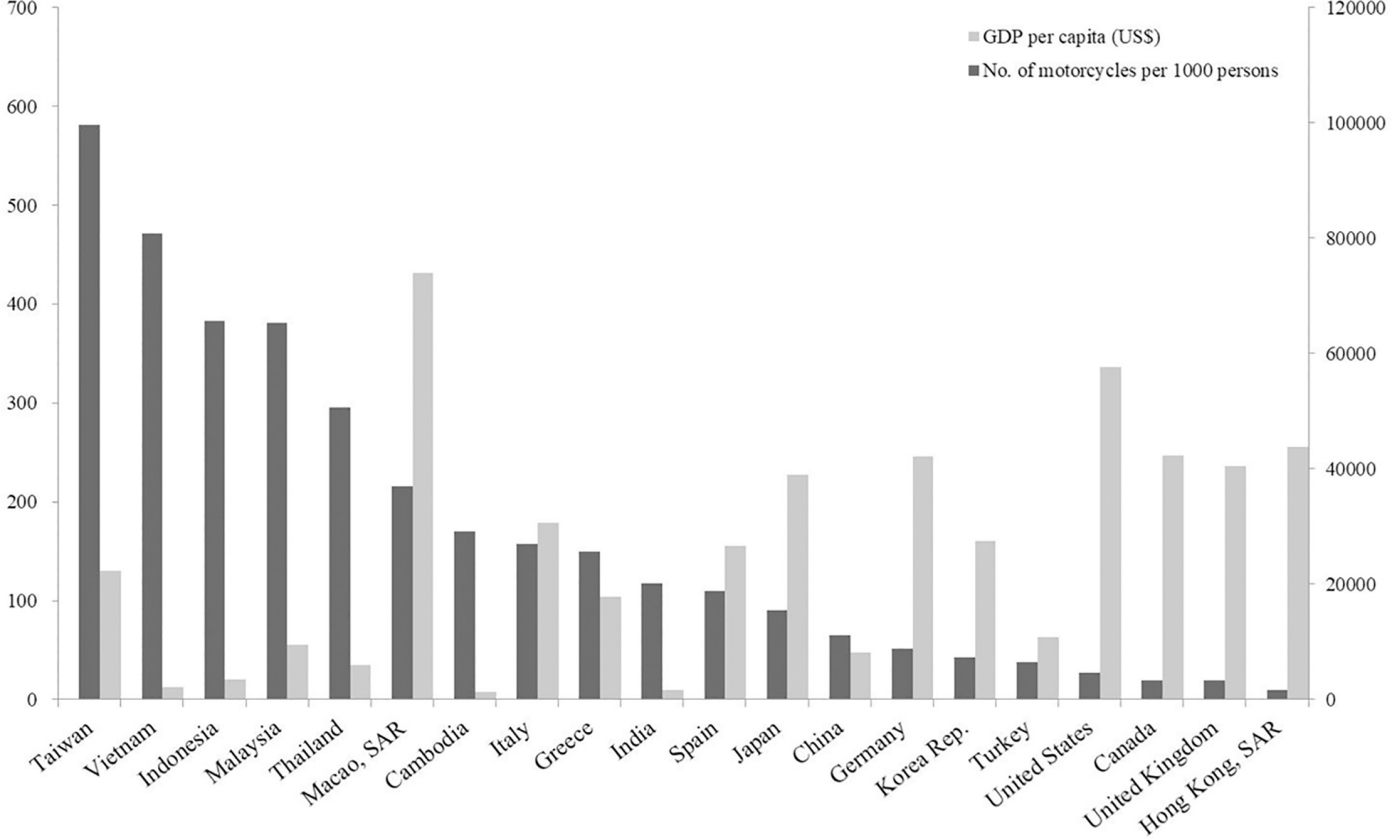
Comparison between the registered motorcycle density and gross domestic product per capita among countries and areas in 2015. GDP, gross domestic product. Data source: S1 Appendix.

Similarly to neighbouring countries, LMCs are widely used in Taiwan for daily commute by students, housewives, and workers. Of note, Taiwan’s motorcycle density is one of the highest in the world (Fig 1). With increasing motorcycle users, 339404 people were reported to be injured due to motorcycle crashes in 2016, and 62.7% of fatalities due to road accidents were caused by motorcycle crashes [3]. This results in a huge burden on emergency care providers’ workload and medical resource consumption. To effectively prevent such injuries, factors affecting injury severity after LMC crashes must be urgently understood.

The assessment of riders’ injury severity through police records was considered to lack consistency and a professional approach [4], and the importance of using hospital data to analyse traffic injury severity has been highlighted recently [5,6]. By adopting hospital data to examine motorcyclist injuries, we attempted to obtain additional variables of rider factors (e.g., helmet type, phone use, riding experience) from a questionnaire survey. These variables were not available from hospital data or police-reported crash data. Although these self-reported data were generally considered subjective, they are still considered a valid approach to collect crucial variables on riding behaviours, which certainly affect motorcyclist injuries [7,8].

The aim of our multicentre study is to determine risk factors for severe injuries of LMC riders in an environment where motorcycles are a mainstream mode of commute, with an emphasis on rider factors, including demographics, comorbidities, and riding behaviours. The data of riders hospitalised after LMC crashes were collected using a structured questionnaire and were linked to hospital data for measuring injury severity. In this study, we used the injury severity score (ISS) [9] and length of hospitalisation (LOH) as injury severity measures.

### Literature review

A literature search of rider factors that affect injury severity after LMC crashes was conducted using the keywords ‘motorcycle crash’, ‘motorcycle accident’, and ‘injury severity’ in PubMed, Scopus, and Google Scholar between 2000 and 2018. We included studies that were conducted in developing countries without a specific mention of motorcycles’ cylinder capacities as well as those conducted in developed countries focusing on LMCs.

Sex and age were previously demonstrated as significant rider factors for injury severity. For instance, male sex was frequently identified as a risk factor [10,11] because men comprise the predominant group of motorcycle users. Elderly riders (aged ≥60 years) were more likely to be severely or fatally injured because of their high comorbidities [12–14]. Inexperience and risky behaviours have been identified as causes of poor outcomes [14–17]. Socioeconomic status was found to affect injury severity [4]. Certain motorcycle makes were reported to be a risk factor [15].

In terms of riding behaviours, drunk riding and using both psychoactive drugs and illicit substances have been globally recognised as crucial factors for motorcyclist casualties [16–19]. Wearing a helmet has been consistently reported to have a protective effect [12,17,19,20]. Although protective clothing can reduce injury severity in riders of heavy motorcycles [21], its effect on LMC crashes has not been investigated. Previous studies have identified risk factors affecting injury severity within patient groups with certain characteristics. Inexperienced riders [17] and obese riders [22] have been reported to have severe injuries. Furthermore, studies have focused on pillion passengers [23], adolescents [24], food delivery motorcyclists [16], and certain injury types [25,26].

Riding speed is widely recognised as a crucial factor. High speed, speed violations, and high road speed limits have been consistently reported to increase injury severity [15–19]. Motorcyclists with more risky behaviours had more severe injuries [17]. Improperly weaving through the traffic and crossing the centre line were associated with severe injuries [16]. Motorcyclists without a valid licence or determined to be at fault had an increased severe injury risk [26]. Lastly, certain precrash behaviours were found to affect injury severity [27].

## Materials and methods

This study was approved by the institutional review boards of participating hospitals (no: N201510012, 16MMHIS168e, J1602, HP17001, and A-ER-105-401). N201510012 for Taipei Medical University (Shuang Ho Hospital and Wan Fang Hospital); 16MMHIS168e for Mackay Memorial Hospital (Taipei, Tamsui, and Taitung Branch); J1602 for Kuang Tien General Hospital; HP17001 for Cheng Ching Hospital Chung Kang Branch; A-ER-105-401 for National Cheng Kung University Hospital.

### Study design and participants

**Fig 2:**
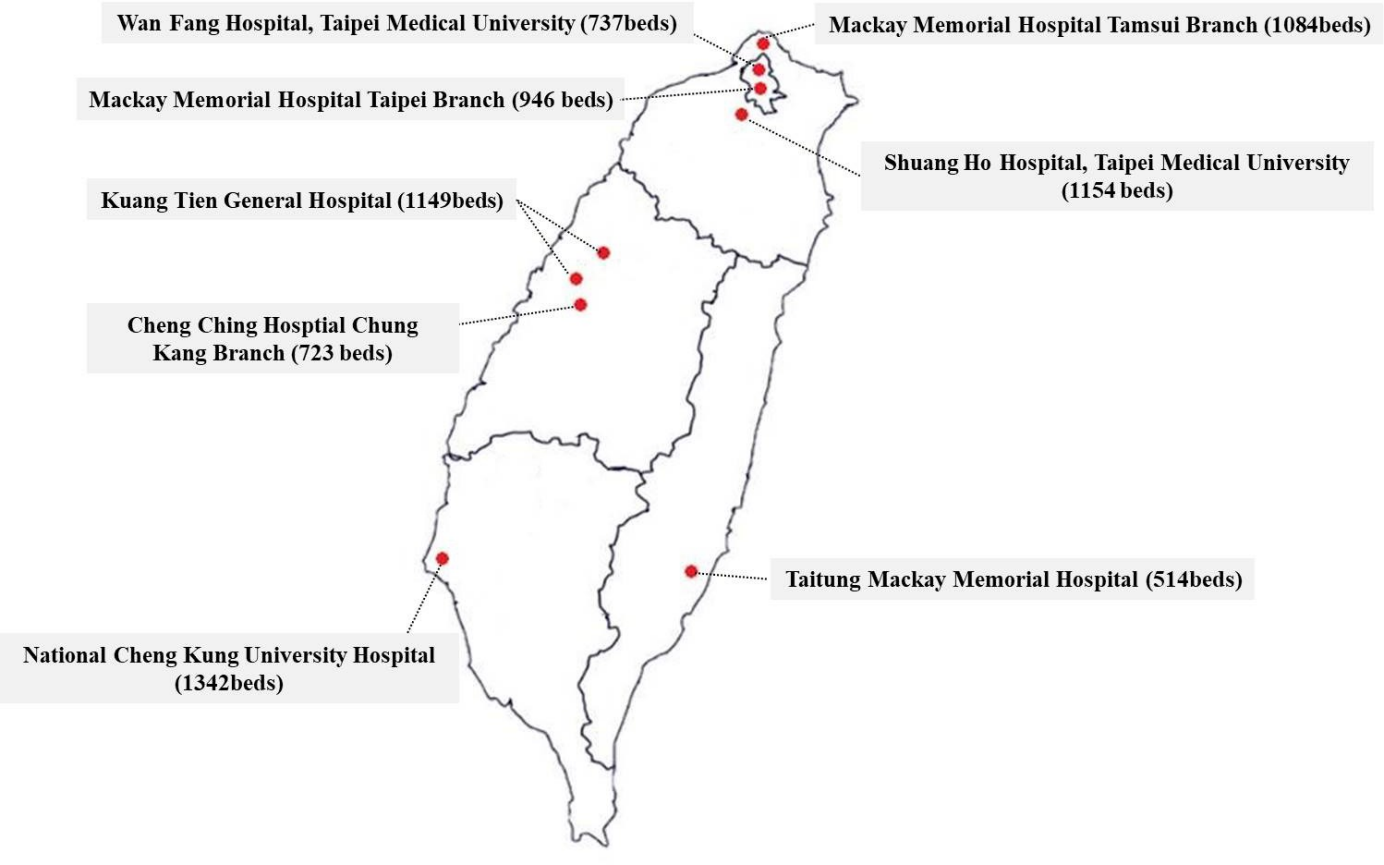
Geographical locations of all participating hospitals.

Taiwan is divided into five districts according to criteria suggested by the National Development Council [28]. Considering the limitations of available resources, we invited at least one hospital from each district (Fig 2). Except Taitung Mackay Memorial Hospital, which is an intermediate emergency responsibility hospital, all other 7 hospitals are advanced emergency responsibility hospitals that can provide comprehensive and sophisticated trauma care.

A list of patients hospitalised within a 2-year interval (2015–2017) before the start of our study was acquired from participating hospitals according to the external cause of motorcycle injury mentioned on patients’ discharge report (International Classification of Diseases, Ninth Revision, Clinical Modification codes E810.2–3, E811.2–3, E812.2–3, E813.2–3, E814.2–3, E815.2–3, E816.2–3, E817.2–3, E818.2–3, and E819.2–3 and International Classification of Diseases, Tenth Revision, Clinical Modification codes V21–V29).

First, we telephonically obtained patients’ consent for participation. Participants were told that they will receive a convenient-store voucher (price: ~US$10) as a token of appreciation for completing the questionnaire and subsequently returning it to us. Next, both consent forms and questionnaires were mailed to patients. Then, data collected from returned questionnaires were typed into a database and linked to the hospital trauma registry by using patients’ personal identification numbers. In our study, we included only gasoline-powered LMC (those with cylinder capacities between 50 and 250 cc) riders aged ≥18 years who could provide consent. The exclusion criterion was being a non-national or not being able to read the questionnaire.

### Questionnaire design

Based on the aforementioned literature review, an expert meeting was conducted for selecting candidate items to construct the questionnaire. Considering the limited space and questions in a postal questionnaire, a Likert scale was used for ranking by experts according to the importance of candidate items, and a questionnaire with 60 questions was finally constructed, including rider factors, such as demographics, comorbidities, and riding behaviours, as well as environmental factors. A pretest was completed by 30 patients, and the content of the questionnaire was revised accordingly (S2 File). To minimize respondents’ self-reporting bias, crosscheck questions were added in our questionnaire. Such a method to filter self-reporting bias was adopted by Guo et al [8]. In Taiwan, the population that consumes caffeinated alcoholic beverages, energy drinks, and betel nuts often overlaps one another. In this study, alcohol and these 3 products were categorised as one variable as ‘alcohol/stimulating refreshment use’ [29,30].

### Statistical analysis

We first conducted a univariate analysis to evaluate the association between each independent variable (potential risk factor) and injury severity measures, including ISS and LOH. The majority of injuries caused by LMC crashes in Taiwan are minor injuries, and morbidity, instead of mortality, is our study’s outcome of interest; thus, we divided ISS into <9 points and ≥9 points and considered ISS ≥ 9 as more severely injured patients [31,32]. LOH was used as a continuous variable for statistical analyses. Log 10-transformation was applied due to the large variation in LOH among the samples, and skew and kurtosis values for the log-transformed LOH had shifted to 0.89 and 1.37, respectively [33]. In terms of independent variables, the chi-squared test was used for categorical variables, and *t*-test was used for continuous variables. Then, a multivariate analysis with stepwise methods was conducted for ISS (Model 1) and LOH (Model 2) by entering independent variables with *P* < 0.2 in a corresponding model of univariate analysis [34].

Considering the dependent variable, ISS, was dichotomous (<9 vs. ≥9 points) in our study, the binary logistic model was used to examine the determinants of severe injuries. The formulation of the binary logistic model [35] is written as follows:

$$g(x)=\beta_{0}+\beta_{1}x_{1}+\beta_{2}x_{2}+\ldots +\beta_{p}x_{p}$$ Eq (1)

where *x*_*j*_ is the value of the *j*th independent variable and *β*_*j*_ as the corresponding coefficient, for *j* = 1, 2, 3…*p*, and *p* is the number of independent variables.

Given the independent variable, the conditional probability of a positive outcome is determined by

$$\pi (x)=\frac{\exp(g(x))}{1+\exp(g(x))}$$ Eq (2)

The maximum likelihood method is employed to measure the associations by constructing the likelihood function as follows:

$$l(\beta )=\prod_{i=1}^{n}\pi {\left(x_{i}\right)}^{y_{i}}{\left(1-\pi (x_{i})\right)}^{1-y_{i}}$$ Eq (3)

where *yi* denotes the *i*th observed outcome, with the value of either 0 or 1, and *i* = 1, 2, 3,…, *n*, where *n* is the number of observations. The best estimate of *β* could be obtained by maximising the log likelihood expression as:

$$LL(\beta )=\ln(l(\beta ))=\sum_{i=1}^{n}\left\{y_{i}\ln(\pi (x_{i}))+(1-y_{i})\ln(1-\pi (x_{i}))\right\}$$ Eq (4)

The effect of attribute on the likelihood of severe injury could be revealed by the odds ratio (OR):

$$OR=\exp(\beta_{j})$$ Eq (5)

with the 95% confidence intervals (CI) of (exp(*β*_*j*_−1.96*sβ*_*j*_), exp(*β*_*j*_ + 1.96*sβ*_*j*_)), where *sβ* is the standard error of the coefficient *β*. An OR that is greater than 1 indicates that the interest attribute leads to a higher probability of an ISS of ≥9. An OR of 1 or close to 1 implies a neutral or weak effect.

Individually, the continuous dependent variable, LOH, was conducted by linear regression model which was usually used to estimate the contribution of the independent variable. A detailed derivation of linear regression model is available in theoretical studies, notably Montgomery et al [36]. SPSS statistical software version 19 (SPSS; Chicago, IL, USA) was used for the analysis, and *P* < 0.05 was considered significant.

## Results

### Respondent profile

**Fig 3:**
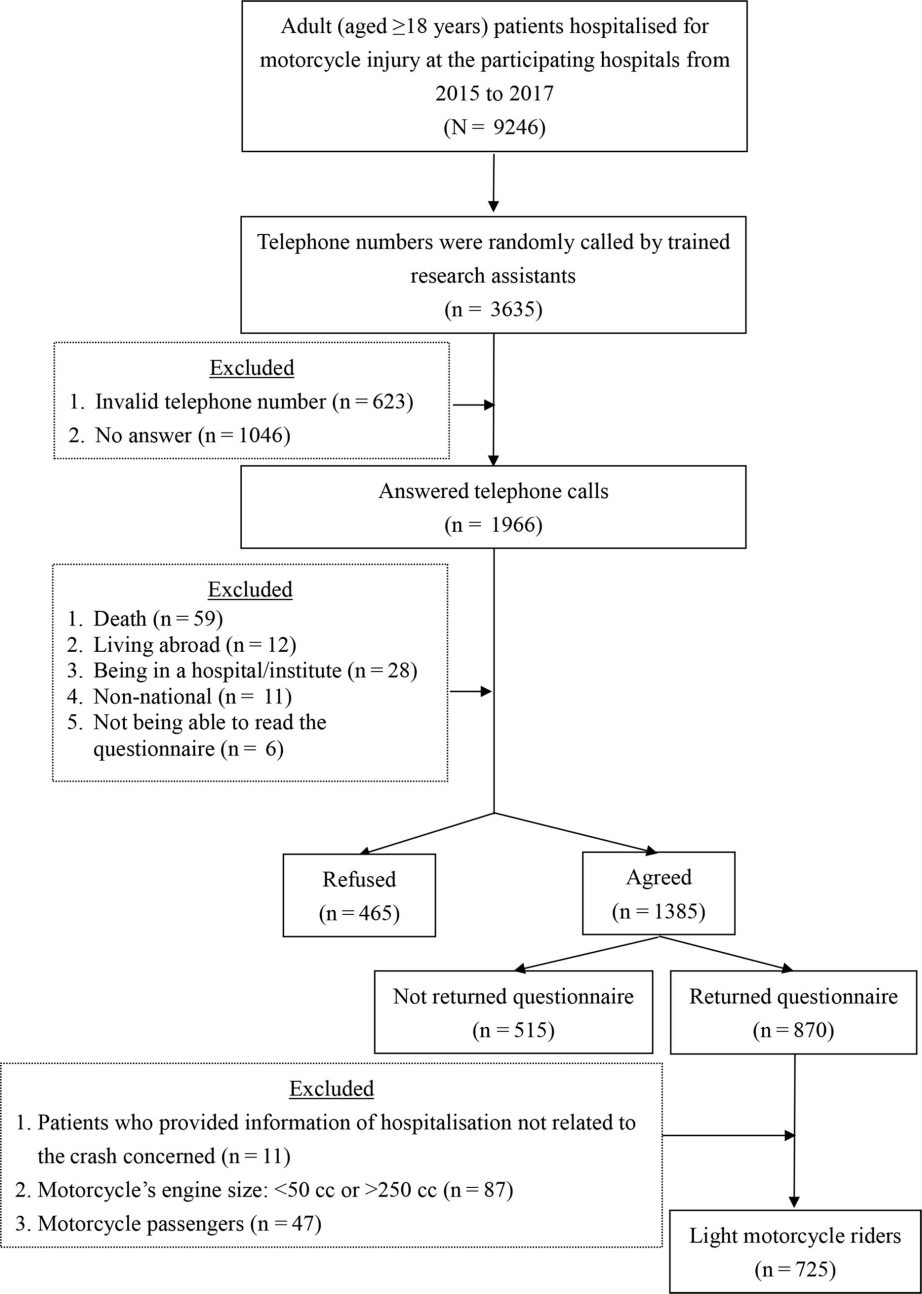
Flow chart for sample selection.

A total of 9246 patients of motorcycle crashes were identified from the eight participating hospitals for the period between 2015 and 2017 (Fig 3). As many as 3635 patients were randomly contacted from an exhaustive list of these 9246 patients. Among these 3635 patients, there were 623 cases with invalid telephone numbers and 1046 cases in which no one answered the phone at all. After removing these cases (n = 1669), 1966 cases remained. Of these 1966 cases in which patients or other family members answered the phones, 59 were found to have passed away, 12 reported to be living abroad, 28 reported to be staying at hospitals, and 6 reported to have difficulties reading the questionnaire. After removing these cases, 1385 patients agreed to participate in the survey and received the questionnaire through post. A total of 870 questionnaires were returned; we excluded the following cases: patients who provided information of hospitalisation not related to the crash concerned (n = 11), patients who reported to be riding motorcycles with an engine size of <50 cc or >250 cc (n = 87), and patients who reported to be motorcycle passengers rather than riders (n = 47). This yielded a sample of 725 patients who were riders of LMCs.

Our study included 464 male patients (64%). The mean age of these participants was 37.73 years. In total, 227 patients (31.31%) were adolescents aged 18–24 years and 65 patients (8.97%) were adults aged ≥65 years. For our sample, the averages of ISS and LOH were 8.24 and 8.3 days, respectively.

### Multivariate analysis

The results of a multivariate analysis between risk factors and an ISS of ≥9 were showed in Table 1. Injury risk with an ISS of ≥9 increased significantly among the patients aged ≥65 years (OR: 3.16, 95% CI: 1.66–6.00). Compared with the patients wearing full-face helmets, the patients wearing half-face helmets were more likely to have an ISS of ≥9 (OR: 1.90, 95% CI: 1.13–3.20). The patients with protective clothing had a higher risk of having an ISS of ≥9 compared with those without protective clothing (OR: 3.58, 95% CI: 1.46–8.77). Among collision objects, heavy truck and car were significant risk factors compared with single-vehicle crashes (OR: 1.92, 95% CI: 1.07–3.45 and 1.69, 1.10–2.61, respectively). Fatigue riding was a risk factor for severe injury (OR: 1.85, 95% CI: 1.07–3.20). By contrast, collisions with an opening door of a parked car resulted in a less severe injury (OR: 0.18, 95% CI: 0.04–0.86). The results of a multivariate analysis between risk factors and LOH was showed in Table 1. Compared with the patients aged 25–44 years, those aged ≥65 years were more likely to have increased LOH (standardised β coefficient: 0.13, 95% CI: 0.05–0.23, *P* = 0.003). Furthermore, encountering motorcycle crashes ≥2 times in the previous year was a significant risk factor (β: 0.11, 95% CI: 0.03–0.18, *P* = 0.007). Regarding comorbidity, the patients with anaemia had increased LOH (β: 0.12, 95% CI: 0.07–0.28, *P* = 0.001). Compared with the patients working in the service industry, those working in commerce had significantly shorter LOH (β: −0.10, 95% CI: −0.16 to −0.01, *P* = 0.026).

**Table 1:** Results of a multivariate analysis between risk factors and severe injury.

	Model 1 (Dependent variable: ISS)				Model 2 (Dependent variable: LOH)				
Variables	OR	95% CI		*P*	*β* coefficient	Standardised *β* coefficient	95% CI		*P*
Age group (year)									
25–44	1.00				Reference				
≥65	3.16	1.66	6.00	0.001	0.14	0.13	0.05	0.23	0.003
Occupation									
Service	-	-	-	-	Reference				
Commercial	-	-	-	-	−0.08	−0.10	−0.16	−0.01	0.026
Frequency of motorcycle crashes in the prior year (time)									
0	-	-	-	-	Reference				
≥2	-	-	-	-	0.10	0.11	0.03	0.18	0.007
Anaemia									
Yes	-	-	-	-	0.17	0.12	0.07	0.28	0.001
Rural crash									
Yes	-	-	-	-	0.06	0.08	0.00	0.11	0.043
Helmet types									
Full face	1.00				Reference				
Half face	1.90	1.13	3.20	0.015	0.07	0.11	0.00	0.14	0.050
Wore protective clothing									
Yes	3.58	1.46	8.77	0.005					
Wore protective boots									
Yes	-	-	-	-	0.14	0.08	0.00	0.27	0.045
Collision objects									
Single-vehicle crashes	1.00				Reference				
Bus/truck	1.92	1.07	3.45	0.030	0.09	0.09	0.01	0.17	0.036
Car	1.69	1.10	2.61	0.017	0.06	0.10	0.00	0.12	0.034
Stationary object	-	-	-	-	0.13	0.08	0.01	0.25	0.037
Crashes with an opening door of parked car									
Yes	0.18	0.04	0.86	0.032	−0.21	−0.10	−0.37	−0.04	0.012
Alcohol/stimulating refreshment use									
Yes	-	-	-	-	0.11	0.08	0.00	0.22	0.042
Fatigue riding									
Yes	1.85	1.07	3.20	0.029	0.08	0.08	0.00	0.16	0.037

Model 1: The dependent variable was ISS, and independent variables included sex and variables with *P* < 0.2 in the univariate analysis. Only significant variables (*P* < 0.05) are shown in Table 1.

Model 2: The dependent variable was LOH, and independent variables included sex and variables with *P* < 0.2 in the univariate analysis. Only significant variables (*P* < 0.05) are shown in Table 1.

CI, confidence interval; ISS, injury severity score; LOH, length of hospitalisation; OR, odds ratio.

Compared with single-vehicle crashes, collisions with a heavy truck, car, or stationary object were risk factors for increased LOH (β: 0.09, 95% CI: 0.01–0.17, *P* = 0.036; 0.10, 0.00–0.12, 0.034; and 0.08, 0.01–0.25, 0.037, respectively). Rural crashes resulted in an increased LOH likelihood (β: 0.08, 95% CI: 0.00–0.11, *P* = 0.043). Crashes with an opening door of a parked vehicle resulted in significantly shorter LOH (β: −0.10, 95% CI: −0.37 to −0.04, *P* = 0.012).

Regarding riders’ behaviours, the patients wearing half-face helmets (compared with full-face ones) had significantly increased LOH (β: 0.11, 95% CI: 0.00–0.14, *P* = 0.050). The patients with protective boots had a higher risk of increased LOH compared with those without protective boots (β: 0.08, 95% CI: 0.00–0.27, *P* = 0.045). Fatigue riding and consumption of alcohol/stimulating refreshments before riding resulted in significantly increased LOH (β: 0.08, 95% CI: 0.00–0.16, *P* = 0.037, and 0.08, 0.00–0.22, 0.042, respectively).

## Discussion

The innovation of the method adopted in this study is twofold. First, we extracted clinical information from the participating hospitals and subsequently administered the questionnaire to obtain additional variables from patients who were discharged from the hospitals. Personal identity numbers and telephone numbers of patients obtained from the participating hospitals were used to track patients and approach individual patients. Such an approach is crucial for obtaining information on important variables, including riding behaviours and helmet types, which is generally not available from both hospital and police-reported data. Second, results derived from the current research may represent the whole population more adequately than those of other studies. A total of eight participating hospitals from five geographic districts in Taiwan provided clinical data. Compared with other studies, such as those conducted by Liang et al. [24] and Liu et al. [22], that were based on a single Level-I hospital in Taiwan, our results are more representative of the population.

Although our method of data collection is certainly innovative, it requires a substantial amount of administrative efforts, research grants, and manpower. For instance, it is challenging and time consuming to obtain approval from the relevant institutional research board of each participating hospital. In addition, another major impediment to collect data from individual patients was patients’ concern over their own privacy when approached by research assistants through telephones. We overcame this problem and increased patients’ willingness to participate in the survey by providing them with a convenient-store voucher.

Regarding rider factors, the results of the multivariate analysis of ISS revealed that the elderly patients were more prone to serious injuries; the same trend was observed for LOH, indicating that the consumption of medical resources increases with age. Previous studies reported that young riders had the highest risk of severe injuries [37]. However, this finding is mostly applicable for motorcyclists in developed countries where motorcycle riding is primarily for recreational or touring purposes [38]. In Taiwan, LMCs are mainly used for daily commute, thus exhibiting a diverse age range of riders. Because of habits established since Taiwan’s rapid economic development between 1970 and 1980, many senior citizens continue to use LMCs for daily commute. Compared with young and middle-aged adults, older adults are more prone to severe injuries because of deteriorations in both cognitive and physical responses to external threats. Furthermore, older adults often have chronic diseases and polypharmacy that result in a high consumption of medical resources during hospitalisation [13]. Currently, Taiwan is an emerging economy with an aging population where many elderly citizens still rely heavily on LMCs. Special attention should be paid to reduce LMC usage among elders and their injury severities in order to reduce subsequent family burden and social loss.

In Taiwan, the frequency of repeated motorcycle crashes is alarming. One and more than one motorcycle crash in the previous year were reported by 16.02% and 10.22% of the participants, respectively. The hospitalised riders who reported more than one motorcycle crash in the previous year were more likely to experience longer LOH than did those who did not. Except the effects of drugs and alcohol, some trauma patients exhibited an injury proneness due to personal characteristics [39]. For instance, riders with an undercontrolled personality were prone to being involved in crashes because their riding behaviors were affected by their tendency of aggressiveness and lack of self-control [40]. Furthermore, repeated injuries may impair patients’ physical functions, resulting in an increased opportunity of recurrent injury [41]. We believe that early identification of the injury-prone group, followed by training provision and monitoring, would reduce their frequency of repeated injuries.

Anaemia considerably increases patients’ LOH. Anaemia is recognised as not only a biomarker of certain chronic diseases [42,43] but also impairment of patients’ physical conditions to cope with injuries [44]. In Taiwan, widespread LMC use among both middle-aged and old-age populations might be the reason for anaemia being identified as a risk factor for severe injuries, because riders in these age groups often have certain chronic diseases. By contrast, heavy motorcycle riders in developed countries tend to be younger. An intervention point to reduce injury risks may include mandatory screening of certain chronic diseases. For example, we found that the anemic patients had longer LOH; as a result, regular screening of the haemoglobin level in patients with anaemia can potentially be an appropriate countermeasure.

Helmets can prevent both severe disability and mortality due to traumatic brain injury [17]. Since 1996, motorcycle helmet use has been compulsory in Taiwan, and 92% of motorcyclists use a helmet while riding. Three helmet types (i.e. full face, open face, and half face) were compared in our study; our results revealed that riders wearing half-face helmets were more prone to higher ISS and longer LOH than did riders wearing full-face helmets. Although head protection has been reported to be insufficient [19,45], breathable half-face helmets are currently prevalent because of the local hot and humid subtropical climate in Taiwan. Although full-face helmets provide the maximum protection, factors such as inconvenience for the wearer, low breathability, and higher costs explain why only 17% of the participants in our study wore full-face helmets. The implementation of compulsory motorcycle helmets has considerably reduced both brain injuries and mortality [45]. To further reduce injury severity after LMC crashes, the public should be educated to wear helmets offering high protection, and lightweight half-face helmet use must be discouraged.

Our study results showed that protective devices, including clothing and boots, were risk factors for higher ISS and longer LOH. Although these protective devices are vital for heavy motorcycle riders’ safety [17], LMC riders in developing countries rarely wear such equipment when commuting on traffic-crowded streets. LMC riders who wear these devices are likely to ride at a high speed. Riders often perceive themselves as safe when wearing protective equipment. However, speeding on regular roads, where motorcycles and other vehicles mix together [46] and road intersections are easily overlooked [47], may lead to severe injuries once a crash occurs.

Unexpected road conditions, such as potholes and foreign objects on road surfaces, insufficient lighting, and road users’ violation of traffic regulations, may put LMC riders at risk. Such conditions are more evident in economically less developed areas. Thus, riders must remain vigilant at all times to prevent accidents. Our study results found that fatigue riding is a significant risk factor for higher ISS and longer LOH [48]. Our results revealed that injuries caused by LMC crashes related to alcohol/stimulating refreshment consumption require longer LOH. Alcohol has been well linked with serious traffic injuries [14,17,18], and its use mixed with energy drinks represents a potential index for risk-taking behaviours [49,50]. Our results showed that alcohol consumption before LMC riding was associated with longer LOH. The current sobriety checkpoint in Taiwan generally targets car drivers but not motorcyclists [51]. To prosecute drunk riding and reduce injury risks, we recommend that the sobriety checkpoint should also target motorcycle riders. However, stimulating refreshments are rarely mentioned as risk factors for severe injuries after motorcycle crashes. In line with the work conducted by O’brien et al. [52] who concluded that college students consuming caffeinated alcoholic beverages had an increased injury risk, we found that riders consuming stimulating refreshments had longer LOH once an accident occurred. Our finding here points to the need for motorcyclist education and campaigns emphasising that simulating refreshments that contain alcohol may impact safety.

Compared with the participants from service industry professions, those from the business sector had shorter LOH. Studies have reported that up to 60% of Taiwanese parents used a motorcycle daily for accompanying their children between home and school [53], and people’s transportation choice is heavily influenced by what their parents use [54]. In addition to their parents’ influence, high degrees of agility and parking convenience attract people to use LMCs. Furthermore, LMCs are preferred as they offer the fastest means of road transportation in urban areas [55]. Thus, some middle-class Taiwanese who can afford a car still prefer an LMC for daily use, which is the antithesis of other cultures in developed countries. The decreased LOH for these patients may be due to their more favourable financial ability, healthier physical and nutritional status, along with being able to acquire robust medical care after injury. However, our study did not identify such associations for ISS, implying that the socioeconomic advantage affects the postcrash course rather than the severity of injury caused by the crash itself.

With respect to environmental factors, our study results showed that LMC crashes that occur in rural areas required longer LOH. The literature has indicated that countryside roads’ harsh conditions, motorcycle speeds, and riders’ motives all have substantial effects on injury severity [4,17]. However, the association between rural crashes and ISS was found to be insignificant in our study, implying that the convenience to gain medical access and the quality of medical care in rural areas are crucial for patients’ postcrash outcomes [56,57].

Compared with single-vehicle crashes, crashes with larger vehicles (e.g. trucks, buses, and car) resulted in both a high ISS and long LOH [58]. Furthermore, crashes with fixed objects, such as trees, walls, and roadside barriers, resulted in a long LOH [26]. Lin et al indicated that riders colliding with vehicles heavier than the motorcycle sustain more severe injuries compared with those colliding with lighter objects [15]. Due to a lack of motorcycle-exclusive lanes, LMC riders generally use the same lane as vehicles of larger sizes. The crash impact between an LMC and these vehicles can easily cause more severe injury to riders [59]. We believe that segregation of LMCs from other heavier vehicles by providing an exclusive lane to LMC riders can effectively reduce injury severity [60].

Riders of motorcycle crashes caused by colliding with opening car doors are at a risk of falling and being run over by approaching vehicles, which often makes news headlines [61]. However, our study showed that these injured riders had lower ISS and shorter LOH. This result may be because deceased victims and patients who were unable to read the questionnaire due to the sequela of severe injury were not included in this study. Another explanation is that such appalling news headlines may remind car drivers to open door carefully, and the motorcycle riders in outer lanes to ride cautiously, thus resulting in fewer such severe injuries. We believe that further exploration of this issue is essential.

Currently, the adoption of electric motorcycles as a green alternative to gasoline-powered motorcycles is promoted globally. However, infrastructure development and incentive provision, which require adequate national financial support, are essential for consumers to accept this substitution [62,63]. This implies that a longer time interval is necessary for this transformation to occur in countries less developed economically.

In recent years, electric motorcycles have effectively been introduced for commuting in numerous countries, and issues of road traffic safety for these vehicles have emerged because of their popularity [7,64,65]. Because high-performance electric motorcycles that can reach a speed of over 90 km/h are continuously introduced in the market [66,67], electric motorcycles operating in a mixed traffic environment may encounter the same risks as gasoline-powered ones. Therefore we believe that our study can provide useful information for the prevention and control of LMC riders’ injury in an environment where motorcycles are widely used to commute, even considering the popularity of such electric motorcycles.

### Limitations

Because this study acquired rider data by using a questionnaire, the deceased or those unable to read the questionnaire because of a disability caused by severe injuries were excluded. Because old people are hesitant to report personal information, they may decline from answering the questionnaire, possibly resulting in age bias. The ISS data of hospitalised patients were obtained retrospectively, which is less accurate than those obtained prospectively. Furthermore, LOH might be influenced by differences in the health delivery system. However, all hospitals that participated in our study are large-scale hospitals under the National Health Insurance system that covers 99% of the total population. Thus, this factor should only have a minimal effect on LOH.

Although our questionnaire included 60 variables related to injury severity of LMC crashes, some important factors still could not be included into the analysis. For example, studies investigating red-light violation (RLV) by bicyclists [8,68–70] and motorcycles [71,72] have reported that RLV had a significant impact on safety. RLV by electric and racing bicycles appeared to be prevalent, and number plates fitted on these two groups of bicycles can potentially be an effective countermeasure to curb RLV [68,69]. However, in our study, as few as 20 patients reported to be a violator of red lights; therefore, we had too few observations on RLV cases to separate out their individual effects. Our study focused on LMC only; by law in Taiwan, all LMCs are mandated to have number plates. Future studies may consider linking our data with police-reported data that provide information on RLV.

## Conclusions

LMCs are widely used for daily commute in developing countries. Thus, severe injuries due to LMC crashes have a major impact on both national productivity and health resource consumption.

Certain factors are significantly associated with riders’ injury severity and related medical resource consumption. Because of differences in the power output, use, and riding environment, risk factors for severe injuries in LMC crashes are dissimilar from those for heavy motorcycles (cylinder capacities > 250 cc) in developed countries and deserve more attention for injury prevention. Because of many similarities in road traffic conditions and riding purposes in both Taiwan and countries or areas with a high dependency on motorcycles, further in-depth evaluation of significant factors based on this study’s results should yield valuable information for road safety authorities and researchers in those countries to reduce severe injuries caused by gasoline-powered or electric LMC crashes.

## Supporting information

S1 Appendix Information sources for registered motorcycles density and gross domestic product per capita. (DOCX)

S1 File Study dataset. (SAV)

S2 File Copy of questionnaire. (PDF)
