# Supplementary material for: Rider factors associated with severe injury after a light motorcycle crash: A multicentre study in an emerging economy setting
Source: PLoS One. 2019 Jun 28;14(6):e0219132. doi: 10.1371/journal.pone.0219132 (PMC6599117; doi:10.1371/journal.pone.0219132)
Supplement: S1 Appendix — (DOCX) [file pone.0219132.s001.docx]

Appendix. Information sources for registered motorcycles density and gross domestic product per capita.

**Information sources for registered motorcycle number:**

*Cambodia*- OECD/ITF. Road Safety Annual Report 2017. Paris: OECD Publishing, 2017.

*Canada*- Available: https://www150.statcan.gc.ca/t1/tbl1/en/tv.action?pid=2310006701. Accessed: 29 May 2018.

*China, Germany, Hong Kong SAR, Italy, Japan, Korea Republic, United Kingdom, and United States*- Available: https://www.motc.gov.tw/ch/home.jsp?id=60&parentpath=0%2C6&mcustomize=statistics401.jsp. Accessed: 1 June 2018.

*Greece*- Available: https://www.ceicdata.com/en/greece/number-of-motor-vehicles/no-of-motor-vehicles-annual-motorcycles. Accessed: 1 June 2018.

*India*- Available: http://www.mospi.gov.in/statistical-year-book-india/2017/189. Accessed: 29 May 29 2018.

*Indonesia*- Available: https://www.bps.go.id/subject/17/transportasi.html#subjekViewTab4. Accessed: 31 May 2018.

*Macao, SAR-* Available: http://www.dsec.gov.mo/Statistic.aspx?NodeGuid=62579ac9-8061-4ede-b4c2-8e7949764582 Accessed: 8 June 2018.

*Malaysia*- Available: https://www.ceicdata.com/en/malaysia/motor-vehicles-registration/number-of-motor-vehicle-private-motorcycle-total. Accessed: 30 May 2018.

*Spain*- Available: http://www.dgt.es/Galerias/seguridad-vial/estadisticas-e-indicadores/publicaciones/principales-cifras-siniestralidad/2017-2799_Summary_Main_figures_on_road_safety_data_Spain_2016_ACCESIBLE.pdf. Accessed: 4 June 2018.

*Taiwan*-. Available: http://statdb.dgbas.gov.tw/pxweb/dialog/statfile9.asp. Accessed: 28 May 2018.

*Thailand*- Available: https://www.m-society.go.th/article_attach/15943/19073.xls. Accessed: 3 June 2018.

*Turkey*- Available: http://www.turkstat.gov.tr/PreHaberBultenleri.do?id=21600. Accessed: 29 May 2018.

*Vietnam*-Available: https://www.unescap.org/sites/default/files/3.%20%20Vietnam%20National%20Road%20Safety%20Goals%20and%20Action%20Plan%20Opportunities%20and%20Challenges.pdf. Accessed: 30 May 2018.

**Source of number of population:**

*Taiwan*- Available: https://eng.stat.gov.tw/lp.asp?CtNode=6339&CtUnit=1072&BaseDSD=36&mp=5. Accessed: 31 May 2018.

*Other countries*- Available: https://data.worldbank.org/. Accessed: 31 May 2018.

**Source of GDP per capita (US$):**

*Taiwan*- Available: https://eng.stat.gov.tw/point.asp?index=1. Accessed: 31 May 2018.

*Other countries*- Available: https://data.worldbank.org/. Accessed: 31 May 2018.
