# Supplementary material for: Rider factors associated with severe injury after a light motorcycle crash: A multicentre study in an emerging economy setting
Source: PLoS One. 2019 Jun 28;14(6):e0219132. doi: 10.1371/journal.pone.0219132 (PMC6599117; doi:10.1371/journal.pone.0219132)
Supplement: S2 File — (PDF) [file pone.0219132.s003.pdf]

(English)

I. Personal Basic Information

1. Gender ☐ (1)Female ☐ (0)Male

1-1. Your age______

2.Occupation

☐ (1) Armed forces/police officer ☐ (2) Government employee ☐ (3) Education
☐ (4) Industrial worker ☐ (5) Business ☐ (6) Skilled agricultural, forestry, and fishery workers
☐ (7) Freelancer ☐ (8) Service employee ☐ (9) Student
☐ (10) Homemaker ☐ (11) Retiree ☐ (12) Other________
☐ (13) Unemployed ☐ (0) Refuse to answer

2-1. If you have a job, then how much is your average monthly income? _________

3. Within the past 12 months, in how many traffic accidents have you been personally involved?
__________times (not including this time) **【Please answer question 3-1 to 3-3 if you had traffic accidents】**

3-1. I have been hospitalised __________ times due to traffic accidents.

3-2. I have visited the emergency department __________ times due to traffic accidents.

3-3. I have suffered only property damage __________ times due to traffic accidents.

4. Your medical history in the past year.

Have you ever been diagnosed with the following diseases by a physician in the past year?	Options
(1) Auditory disease **(You can choose multiple answers)**<br>（☐deafness ☐Meniere's disease ☐tinnitus ☐ difficulty in hearing ☐other________）	☐No ☐Yes
(2) Visual illnesses **(You can choose multiple answers)**<br>（☐ myopia ☐ presbyopia ☐ cataract ☐ xerophthalmia ☐ other_________）	☐No ☐Yes
(3) Cerebrovascular disease	☐No ☐Yes
(4) Heart disease	☐No ☐Yes
(5) Lung disease	☐No ☐Yes
(6) Diabetes	☐No ☐Yes
(7) Hypertension	☐No ☐Yes
(8) Liver disease	☐No ☐Yes
(9) Gastric ulcer and duodenal ulcer	☐No ☐Yes
(10) Kidney disease	☐No ☐Yes
(11) Cancer	☐No ☐Yes
(12) Epilepsy	☐No ☐Yes
(13) Anaemia	☐No ☐Yes
(14) Osteoporosis	☐No ☐Yes
(15) Arthritis	☐No ☐Yes
(16) Alcoholism	☐No ☐Yes
(17) Parkinson disease	☐No ☐Yes
(18) Mental illness (e.g., depression, anxiety, bipolar disorder)	☐No ☐Yes
(19) Dementia	☐No ☐Yes
(20) Upper limb or lower limb motor dysfunction (including caused by trauma or disease)	☐No ☐Yes

II. Can you explain to us what happened in the traffic accident that hospitalised you?

5. Time of traffic accident: _____ (YYYY) _____ (MM) _____ (DD) ☐ _______AM or ☐ _______PM.

6. Where did the traffic accident took place? **【Answer question 6-1 if it happened at a downtown area. Answer question 6-2 if it happened at a provincial/county highway】**

 6-1. ________ County (City) ________ Township (City, District) at ________ Road (Street) around__________________.

 6-2. Highway No. ___________ at ____ km ____ m heading ___________ (direction) towards ___________ (destination).

7. What was your status when the traffic accident happened?

 ☐ (1) Passenger ☐ (0) Rider

 7-1. When the motorcycle accident occurred: **(You can choose multiple answers)【If you were rider, please skip this question】**

  ☐ (1) I felt that our vehicle was too fast.

  ☐ (2) I felt that our vehicle was violating the red light.

  ☐ (3) I was using mobile phone or something like a handheld device

  ☐ (4) I was talking to the rider.

  ☐ (5) I felt that the motorcycle was slipping.

  ☐ (0) None.

 7-2. What was your (the passenger's) posture when the accident occurred? **(You can choose multiple answers) 【If you were rider, please skip this question】**

  Hands: ☐(1) Holding the rider's waist. ☐(2) Holding the backseat of the vehicle.

  ☐(3) Hands on the rider's shoulder. ☐(4) Not holding/holding tight to anything.

  ☐(5) Other________

  Legs: ☐(1) Straddling with both legs opens. ☐(2) Legs together facing the left.

  ☐(3) Legs together facing the right. ☐(4) Other________

8. Were there passengers on the vehicle?

 ☐ (1) One passenger. ☐ (2) Two or more passengers. ☐ (0) None.

9. Was there any item on the vehicle?

 ☐ (1) Yes, about _______ kg. ☐ (0) None.

10. What was the **road speed limit** at the location of the traffic accident?

 ☐ (1) The road speed limit was _______ km/hr. ☐(0) I was not aware of that.

11. Did you receive any violation tickets due to this traffic accident?

 ☐ (1) Yes, due to _____________. ☐ (0)No.

12. Did you wear a helmet when the traffic accident occurred? If you did, what kind of helmet did you wear?

 ☐ (1) Beanie Helmet ☐ (2) Half Helmet ☐ (3) Open Face ☐ (4) Full Face ☐ (0) None.

13. Besides the helmet, what other protective gear did you wear? **(You can choose multiple answers)**

 ☐ (1) Protection Jacket ☐ (2) Gloves ☐ (3) Boots

 ☐ (4) Guards (shoulder protectors, elbow protectors, or knee protectors) ☐ (0) None.

14. What was the type of motorcycle you rode when the traffic accident occurred?

☐ (1) Green plate ≤ 50 cc ☐ (2) White plate 51–250 cc

☐ (3) Yellow plate 251–550 cc ☐ (4) Red plate ≥ 551 cc

14-1. What was the brand of motorcycle you rode when the traffic accident occurred?

☐ (1) KYMCO ☐ (2) YAMAHA

☐ (3) SYM ☐ (4) Other_____________

15. What was the first collision situation (first point of collision) of the traffic accident? **(Choose one answer)**

☐ (1) Collided with a bus, including city buses, regional buses, tour buses, etc.
☐ (2) Collided with a large truck.
☐ (3) Collided with a trailer.
☐ (4) Collided with a light passenger vehicle.
☐ (5) Collided with a small truck.
☐ (6) Collided with a motorcycle.
☐ (7) Collided with pedestrian(s).
☐ (8) Collided with a bicycle.
☐ (9) Spillover on your own.
☐ (10) Collided with nonmoving objects, such as utility poles, refuge islands, trees, or others.
☐ (11) Collided with an animal.
☐ (12) Collided with a train.
☐ (13) Railway crossing accident (collided with railway crossing fences).
☐ (14) Others (please explain): ____________

16. What were the reasons that you could not evade the traffic accident? **(You can choose multiple answers)**

☐ (1) The other vehicle was too fast.
☐ (2) The other vehicle suddenly changed direction.
☐ (3) The other vehicle suddenly opened the door.
☐ (4) Vehicles, pedestrians, or animals suddenly appeared.
☐ (5) The signal light suddenly changed.
☐ (6) Felt that I was riding too fast.
☐ (7) Felt that I suddenly changed direction.
☐ (8) I drifted off.
☐ (9) Slippery road while raining.
☐ (10) Poor visibility.
☐ (11) Others: **【Please write down the possible reasons】** ____________________________

17. We wish to understand your status **before you rode**:

17-1. Did you take any medicine before riding? **(You can choose multiple answers)**

☐ (1) Sleeping pills ☐ (2) Cold medicines

☐ (3) Medicines for heart diseases ☐ (4) Medicines for psychiatric disorders

☐ (5) Others: _______________ ☐ (0) None

17-2. Did you drink alcohol or take any refreshments before riding? **(You can choose multiple answers)**

☐ (1) Alcohol. ☐ (2) Betel nuts. ☐ (3) Coffee.

☐ (4) Refreshment drinks besides coffee, such as ___________ ☐ (0) None.

18. We wish to understand the situation **when the accident happened**:

18-1. What was your **speed**?

☐ (1) _______km/h ☐ (0) I am not sure.

18-2. What was your physical and mental status? **(You can choose multiple answers)**

☐ (1) I drifted off.

☐ (2) I was thinking about something else.

☐ (3) I felt tired.

☐ (4) My body was uncomfortable.

☐ (5) I was in a poor mood.

☐ (6) Others:**【Please write down your physical and mental status when the accident occurred】** ____________________________

☐ (0) Normal.

18-3. Were you doing the following things while riding? **(You can choose multiple answers)**

☐ (1) Using mobile phone or tablet PC. **【Please choose from the following: ☐Talking on the phone; ☐ Sending text message; ☐ Navigating; ☐ Listening to music; ☐ Playing games and others】**

☐ (2) Finding my way (looking for a certain address, shop, or parking space).

☐ (3) Eating while riding.

☐ (4) Talking to the passenger.

☐ (5) Taking care of a pet or child.

☐ (6) Paying attention to an item on the vehicle or looking for something.

☐ (7) Others: **【Please write down what you did】** ____________________________

☐ (0) None.

18-4. Were you distracted by any of the following situations that might have caused the traffic accident? **(You can choose multiple answers)**

☐ (1) Looking at the scenery or activities (such as events held by shops or other car accidents).

☐ (2) There were dazzling lights or it was raining, which obstructed your view.

☐ (3) There were vehicles or obstacles in front or by the road so that you could not see what happened in front.

☐ (4) Others **【Please write down what happened】:** ____________________________

☐ (0) None.

III. With the following questions, we want to understand your situation after you were hospitalised and receiving treatments due to the traffic accident (all questions here refer to the effects caused by hospitalisation due to this traffic accident, not including the effects caused by previous accidents).

19. Did you accept treatments from long-term care institutes after leaving the hospital?

☐ (1)Yes. ☐ (0)No.

20. Did you apply for "Catastrophic Illness Card" for the treatment?

☐ (1)Yes. ☐ (0)No.

21. Were your family or friends with you or did you hire a nursing worker to take care of you?

☐ (1) Yes. They were with me for ________ days. ☐(0)No.

22. Besides medical expenses, what other expenses did you pay due to this traffic accident?

(1) Vehicle damage: NT$_________
(2) Item damage: NT$_________
(3) Compensation: NT$_________
(4) Private insurance claim: NT$_________
(5) Others: NT$_________

IV. Due to your past driving behavior and experience, please give a mark to the following situations from 1 (never happened) to 6 (happened almost every time).

Definition of frequency (for reference)<br>Never happened: none within one year<br>Seldom happened: once within six months<br>Sometimes happened: once within three months<br>Often happened: once within one month<br>Usually happened: once within one week<br>Happened almost every time: almost happened when you riding	Never happened(1)	Seldom happened(2)	Sometimes happened (3)	Often happened(4)	Usually happened	Happened almost every time (6)
23. When turning into the arterial road that is having traffic congestion, you pay too much attention to the traffic flow of the arterial road and nearly collide into the car in front of you.	☐	☐	☐	☐	☐	☐
24. You pay attention to street-crossing pedestrians when turning into a lane.	☐	☐	☐	☐	☐	☐
25. You honk the horn to other road occupants to show your dissatisfaction.	☐	☐	☐	☐	☐	☐
26. You look into the side mirrors before changing lanes.	☐	☐	☐	☐	☐	☐
27. You hit the brake abruptly on slippery roads or turn the handlebar into the wrong direction when you slip.	☐	☐	☐	☐	☐	☐
28. When turning at the intersection, you force vehicles going straight to make way for you to go first.	☐	☐	☐	☐	☐	☐
29. You ignore the speed limit at residential areas.	☐	☐	☐	☐	☐	☐
30. When turning right, you nearly have slight impact with bicycles coming from your right rear side.	☐	☐	☐	☐	☐	☐
31. Miss “Give Way” signs and nearly collide into other vehicles or pedestrians.	☐	☐	☐	☐	☐	☐
32. When you attempt to overtake another vehicle, you forget to use turn signals.	☐	☐	☐	☐	☐	☐
33. When you are agitated by other drivers, you chase that driver to show your anger.	☐	☐	☐	☐	☐	☐
34. When encountering narrowed roadways, you only change the lane at the last minute.	☐	☐	☐	☐	☐	☐
35. Overtake slow moving vehicles driving/riding at the inside lanes.	☐	☐	☐	☐	☐	☐
36. You want to surpass other vehicles and ride quickly in front of traffic lights.	☐	☐	☐	☐	☐	☐
37. You stay too close to the vehicle in front you so that you can hardly hit the brake in case of emergency.	☐	☐	☐	☐	☐	☐
38. You run the lights at intersections.	☐	☐	☐	☐	☐	☐
39. When being agitated by the behaviors of other drivers, you use all means to express your anger.	☐	☐	☐	☐	☐	☐
40. When overtaking another vehicle, you underestimate the speed of the vehicles coming from the opposing lane.	☐	☐	☐	☐	☐	☐
41. You follow the speed limit on roads.	☐	☐	☐	☐	☐	☐
42. You ride the wrong way due to convenience.	☐	☐	☐	☐	☐	☐
43. When you ride at the outside lane and encountered parking buses or cars, you	☐	☐	☐	☐	☐	☐

ride into the "No Motorcycle Lane" or on the sidewalks.						
44. When encountering hook turns, you just turn left to save trouble.	☐	☐	☐	☐	☐	☐
45. You do not use turn signals when turning directions.	☐	☐	☐	☐	☐	☐
46. You stay close behind or near large vehicles.	☐	☐	☐	☐	☐	☐
47. In order to save time, you pass through narrow spaces between vehicles or by the side of other vehicles.	☐	☐	☐	☐	☐	☐
48. You check the wear condition of your tires before riding.	☐	☐	☐	☐	☐	☐
49. You check if the headlight, turn signals, and brake lights are functioning normally or not before riding.	☐	☐	☐	☐	☐	☐
50. You talk with the passenger when riding.	☐	☐	☐	☐	☐	☐

V. Measurement on QOL (Eq-5D). Please check one situation that best fits your health today from the following questions.

1. **Mobility**
   - ☐ I have no problems in walking about
   - ☐ I have slight problems in walking about
   - ☐ I have moderate problems in walking about
   - ☐ I have severe problems in walking about
   - ☐ I am unable to walk about
2. **Self-Care**
   - ☐ I have no problem washing or dressing myself
   - ☐ I have slight problems washing or dressing myself
   - ☐ I have moderate problems washing or dressing myself
   - ☐ I have severe problems washing or dressing myself
   - ☐ I am unable to wash or dress myself
3. **Usual Activities** (e.g. work, study, housework, family or leisure activities)
   - ☐ I have no problem doing my usual activities
   - ☐ I have slight problem doing my usual activities
   - ☐ I have moderate problem doing my usual activities
   - ☐ I have severe problem doing my usual activities
   - ☐ I am unable to do my usual activities
4. **Pain/Discomfort**
   - ☐ I have no pain or discomfort
   - ☐ I have slight pain or discomfort
   - ☐ I have moderate pain or discomfort
   - ☐ I have severe pain or discomfort
   - ☐ I have extreme pain or discomfort
5. **Anxiety/Depression**
   - ☐ I am not anxious or depressed
   - ☐ I am slightly anxious or depressed
   - ☐ I am moderately anxious or depressed
   - ☐ I am severely anxious or depressed
   - ☐ I am extremely anxious or depressed

VI. So far (today), how do you evaluate your overall health status?

Best imaginable
state of health

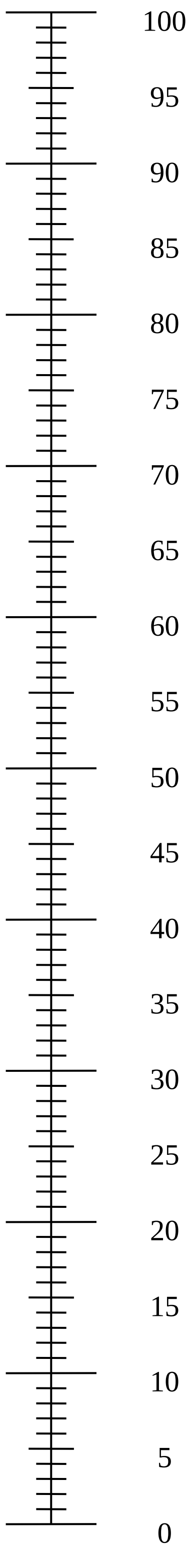

Worst imaginable
state of health

- We would like to know how good or bad your health is TODAY.
- This scale is numbered from 0 to 100.
- 100 means the <u>best</u> health you can imagine.
- 0 means the <u>worst</u> health you can imagine.
- Mark an X on the scale to indicate how your health is TODAY.
- Now, please write the number you marked on the scale in the box below.

YOUR HEALTH TODAY = ☐

(Original language)

一、個人基本資料

1.您的性別是 □(1)女 □(0)男

1-1.您的年齡是______歲

2.請問您的職業類別為？

□(1)軍、警 □(2)公 □(3)教
□(4)工 □(5)商 □(6)農、林、漁、牧
□(7)自由業 □(8)服務業 □(9)學生
□(10)家管 □(11)退休 □(12)其他________
□(13)無業/待業/失業中 □(0)拒答

2-1.若有工作，請問您的平均月收入約__________元

3.請問在**本次交通事故前**的 12 個月間，您本人曾經發生過多少次交通事故？______次**【不包含本次】**

【若有發生交通事故請接著填寫 3-1～3-3 題】

3-1.因交通事故而住院：__________次

3-2.因交通事故只到急診治療未住院：__________次

3-3.無受傷僅車損傷：__________次

4.過去一年的病史

過去一年內您是否曾經被醫師診斷過下列病症？	選項
(1) 聽覺方面的疾病（**可複選**）<br>（□失聰、□梅尼爾氏症、□耳鳴、□重聽、□其他________）	□否 □是
(2) 視覺方面的疾病（**可複選**）<br>（□近視、□老花、□白內障、□乾眼症、□其他________）	□否 □是
(3) 腦血管疾病	□否 □是
(4) 心臟疾病	□否 □是
(5) 肺臟疾病	□否 □是
(6) 糖尿病	□否 □是
(7) 高血壓	□否 □是
(8) 肝臟疾病	□否 □是
(9) 胃潰瘍、十二指腸潰瘍	□否 □是
(10)腎臟疾病	□否 □是
(11)癌症	□否 □是
(12)癲癇	□否 □是
(13)貧血	□否 □是
(14)骨質疏鬆	□否 □是
(15)關節炎	□否 □是
(16)酒精中毒	□否 □是
(17)巴金森氏症	□否 □是
(18)精神疾病（憂鬱症、焦慮症、躁鬱症等）	□否 □是
(19)失智症	□否 □是
(20)上肢或下肢運動功能障礙（包括外傷或疾病造成）	□否 □是

二、請您回憶一下造成您本次住院的交通事故，發生當時的狀況：

5.請問您發生交通事故的時間是_____年_____月_____日 □上午_______時或 □下午_______時

6.您發生交通事故的地點在**【如地點在市區請填 6-1 題，在省/縣道請填 6-2 題】**

6-1. ________縣 (市)________鄉 (鎮、市、區)________路 (街)約在_________________的位置

6-2. 路線及里程編號__________公路____公里____公尺處__________向__________車道

7.請問發生交通事故當時您的身分為？

□ (1)乘客　　□ (0)駕駛

7-1.請問您(乘客)事故發生當時：**(可複選)【若為駕駛，請略過此題】**

□ (1)感覺我方車速過快

□ (2)感覺我方有闖紅燈

□ (3)我有使用手機之類的 3C 產品

□ (4)和駕駛聊天

□ (5)感覺車輛打滑

□ (0)無

7-2.請問事故發生時，您(乘客)乘坐的姿勢。**(可複選)【若為駕駛，請略過此題】**

手部姿勢：□(1)手抱駕駛腰部　□(2)手抓機車後座　□(3)手搭駕駛肩膀

□(4)手未抓(牢)　□(5)其他________

腳部姿勢：□(1)雙腿分開跨坐　□(2)雙腿朝左邊側坐　□(3)雙腿朝右邊側坐

□(4)其他________

8.請問當時車上是否有載人？

□(1)載 1 人　□ (2)載 2 人或 2 人以上　□ (0)沒載人

9.請問當時車上是否有載物品？

□ (1)有，約_______公斤　□ (0)無

10.請問您發生交通事故當時，**道路速限**是多少？

□(1)道路速限_______公里　□(0)不清楚

11.請問您這次交通事故有收到罰單嗎？

□ (1)有 原因_____________　□ (0)無

12.請問您在這次交通事故發生時是否有戴安全帽，請選擇其中一項？

□ (1)半罩式　□(2)半露臉式　□ (3)露臉式　□ (4)全面式　□ (0)無

13.除了安全帽外，當時您還有穿戴哪些防護具？**(可複選)**

□ (1)防護夾克　□ (2)手套　□(3)靴子　□(4)護甲(護肩、護肘、護膝)　□ (0)無

14.請問發生交通事故當時所使用的機車車種為何？

□ (1)綠牌 ≤50cc　□ (2)白牌 51cc~250cc　□ (3)黃牌 251cc~550cc　□ (4)紅牌 ≥551cc

14-1.請問您發生交通事故當時所使用的機車廠牌為何？

□ (1)光陽　□ (2)山葉

□ (3)三陽　□ (4)其他____________

15.請問交通事故發生時最先的（第一個碰撞點）碰撞情況如何？**（單選）**

□ (1)與大客車發生碰撞，如公車、客運、遊覽車等。
□ (2)與大貨車發生碰撞
□ (3)與聯結車發生碰撞
□ (4)與小客車發生碰撞
□ (5)與小貨車發生碰撞
□ (6)與機車發生碰撞
□ (7)與行人發生碰撞
□ (8)與自行車發生碰撞
□ (9)自摔
□ (10)與非移動物體發生碰撞，如電線桿、安全島、路樹等。
□ (11)與動物發生碰撞
□ (12)與火車發生碰撞
□ (13)平交道事故(衝撞平交道柵欄)。
□ (14)其他 (請說明)：____________

16.交通事故發生時您來不及閃避的原因是？**（可複選）**

□ (1)對方車輛速度過快
□ (2)對方車輛突然變換方向
□ (3)對方車輛突然開車門
□(4)突然冒出的車輛、行人或動物
□ (5)紅綠燈燈號突然變換
□ (6)感覺自己騎車速度過快
□ (7)感覺自己騎車變換方向太快
□ (8)恍神
□ (9)天雨路滑
□ (10)視線不佳
□ (11)其他：**【請寫出可能的原因】**____________________________

17.希望能瞭解您在**騎車之前**的狀況：

17-1.請問您在騎車之前是否有在服用藥物？**（可複選）**

□ (1)安眠藥　□ (2)感冒藥　□ (3)心臟科用藥
□ (4)精神科用藥　□ (5)其他：_______________　□ (0)無

17-2.請問您在騎車之前是否有飲酒或吃其他提神物？**（可複選）**

□ (1)有飲酒　□ (2)有嚼檳榔　□ (3)有飲用咖啡
□ (4)有飲用咖啡以外的提神飲料:例如___________　□ (0)無

18.希望能瞭解您**發生事故當時**的狀況：

18-1.請問您當時的**行車速度**大概多少？

□ (1)行車時速_______公里　□(0)不清楚

18-2. 請問在發生事故前，您的身體及心理是否有感受到下列的狀況？**（可複選）**

□ (1)恍神
□ (2)想事情
□ (3)疲勞、累
□ (4)身體不舒服
□ (5)情緒差
□ (6)其他：**【請寫出事故當時身體和心理感受到的其他狀況】**__________________________
□ (0)正常

18-3.請問您是否有邊騎車，邊做以下事情？（**可複選**）

☐ (1)有使用手機或平板【**請勾選□通電話、□傳訊息、□導航、□聽音樂、□玩遊戲等**】

☐ (2)邊找路（找地址、店家、停車位等）

☐ (3)邊吃東西

☐ (4)和乘客聊天

☐ (5)照顧寵物或小孩

☐ (6)注意車上搭載的物品或找東西

☐ (7)其他：【**請寫出所做的事情**】___________________________

☐ (0)無

18-4.請問當時周遭有無下列使您分心的狀況，導致您發生車禍？（**可複選**）

☐ (1)觀看路旁的風景或人群活動（如：商店舉辦活動、車禍等）

☐ (2)過於刺眼的光線或下雨等，讓您視線受到影響

☐ (3)前方或路旁有車輛或障礙物，讓您一時無法看清楚前方狀況

☐ (4)其他【**請寫出所做的事情**】: ___________________________

☐ (0)無

三、以下題目是想瞭解您因為這次交通事故接受住院治療的狀況（**所有題目內容皆指這次住院的交通事故對您造成的影響，不包含過去意外事故造成的影響**）

19.請問您出院後是否入住長期照護機構接受後續的照護？

☐ (1)有　　　☐ (0)無

20 請問您這次治療是否有申請重大傷病卡？

☐ (1)有　　　☐ (0)無

21.請問您這次住院是否有親友或聘請看護陪伴？

☐ (1)有，總共陪伴________天　　　☐(0)無

22.除了上述提到的費用外，您因為這次交通事故大約支付了多少其他的費用？

(1) 車輛毀損：_________元

(2) 物品毀損：_________元

(3) 賠償金：_________元

(4) 私人保險理賠金：_________元

(5) 其他：_________元

四、請就您過去的駕駛行為經驗，依照發生頻率給予 1（未曾發生）到 6（幾乎每次）的評分

發生頻率的定義 (供參考)<br>未曾發生：一年沒有一次<br>幾乎沒有：半年一次<br>有時這樣：三個月一次<br>經常如此：一個月一次<br>常常這樣：一週一次<br>幾乎每次：幾乎每次騎車都會發生	未曾發生(1)	幾乎沒有(2)	有時這樣(3)	經常這樣(4)	常常這樣(5)	幾乎每次(6)
23. 轉入塞車的主幹道時，因為太注意主幹道上的車流而差點撞到前車	☐	☐	☐	☐	☐	☐
24. 轉入巷子時會注意到過馬路的行人	☐	☐	☐	☐	☐	☐
25. 向其他用路人按喇叭表示您的不滿	☐	☐	☐	☐	☐	☐
26. 變換車道前會看後照鏡	☐	☐	☐	☐	☐	☐
27. 您行駛在濕滑路面時會急煞，或在打滑時機車龍頭轉錯方向	☐	☐	☐	☐	☐	☐
28. 在交叉路口準備轉彎時，您會逼直行車讓路，讓您先行	☐	☐	☐	☐	☐	☐
29. 忽略住宅區的速限	☐	☐	☐	☐	☐	☐
30. 右轉時差點與右後方過來的自行車發生擦撞	☐	☐	☐	☐	☐	☐
31. 未注意「讓」字標誌，差點撞上其他車輛或行人	☐	☐	☐	☐	☐	☐
32. 嘗試超車，但忘記要打方向燈	☐	☐	☐	☐	☐	☐
33. 被其他駕駛激怒後，為了表明您的憤怒，您會追逐該駕駛	☐	☐	☐	☐	☐	☐
34. 遇到道路縮減時，您會在最後一刻才勉強變換車道	☐	☐	☐	☐	☐	☐
35. 對行駛在內側車道的慢速車超車	☐	☐	☐	☐	☐	☐
36. 紅燈轉綠燈前，您會為了超越其他車輛而快速衝出去	☐	☐	☐	☐	☐	☐
37. 太靠近前車，以至於緊急狀況時難以煞車	☐	☐	☐	☐	☐	☐
38. 您會在交叉路口闖紅燈	☐	☐	☐	☐	☐	☐
39. 被其他駕駛的行為激怒後，會用任何手段表明你的憤怒	☐	☐	☐	☐	☐	☐
40. 超車時低估對向來車的速度	☐	☐	☐	☐	☐	☐
41. 在道路上會遵守速限	☐	☐	☐	☐	☐	☐
42. 為了方便而逆向行駛	☐	☐	☐	☐	☐	☐
43. 在外側車道行駛遇有停靠公車與汽車時，會行駛禁行機車道或駛上人行道	☐	☐	☐	☐	☐	☐
44. 遇有機車兩段式左轉管制時，會因覺得麻煩而直接左轉	☐	☐	☐	☐	☐	☐
45. 在轉彎時，沒有使用方向燈	☐	☐	☐	☐	☐	☐
46. 會緊跟在大型車的後面或附近	☐	☐	☐	☐	☐	☐
47. 為了快點，會從其他車輛旁的極小空間通過	☐	☐	☐	☐	☐	☐
48. 騎車前會檢查輪胎的磨損	☐	☐	☐	☐	☐	☐
49. 騎車前會檢查頭燈、方向燈與煞車燈作用是否正常	☐	☐	☐	☐	☐	☐
50. 騎車時會與乘客聊天	☐	☐	☐	☐	☐	☐

五、生活品質量表（EQ-5D），請在每個題目下，勾選一個最能描述您今天健康狀況的情形。

1. 行動能力

□我四處走動沒有困難

□我四處走動有一點困難

□我四處走動有中度的困難

□我四處走動有嚴重的困難

□我無法四處走動

2. 自我照顧

□我自己洗澡或穿衣沒有困難

□我自己洗澡或穿衣有一點困難

□我自己洗澡或穿衣有中度的困難

□我自己洗澡或穿衣有嚴重的困難

□我無法自己洗澡或穿衣

3. 日常活動（如工作、讀書、家事、家庭或休閒活動）

□我進行日常活動沒有困難

□我進行日常活動有一點困難

□我進行日常活動有中度的困難

□我進行日常活動有嚴重的困難

□我無法進行日常活動

4. 疼痛/ 不舒服

□我沒有疼痛或不舒服

□我有一點疼痛或不舒服

□我有中度的疼痛或不舒服

□我有嚴重的疼痛或不舒服

□我有非常嚴重的疼痛或不舒服

5. 焦慮 / 沮喪

□我沒有焦慮或沮喪

□我有一點焦慮或沮喪

□我有中度的焦慮或沮喪

□我有嚴重的焦慮或沮喪

□我有非常嚴重的焦慮或沮喪

六、您目前(今天)對自己整體健康狀況的評估為何？

您想像中最好
的健康狀況

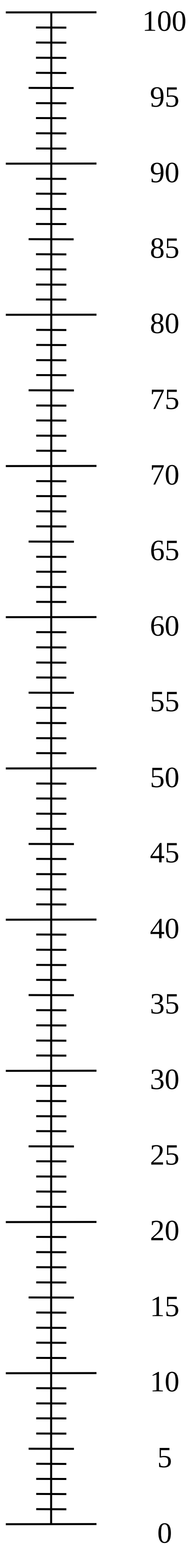

您想像中最差
的健康狀況

- 我們想知道您**今天**健康狀況的好壞。
- 這個刻度尺有從 0 到 100 的數字。
- 100 代表您想像中<u>最好</u>的健康狀況。
- 0 代表您想像中<u>最差</u>的健康狀況。
- 請在刻度尺上打個 ”X”，指出您**今天**的健康狀況。
- 並請在以下空格中，寫下您在刻度尺上標示的那個數字。

您今天整體的健康狀況 = ☐
